# Google trends as an early indicator of African swine fever outbreaks in Southeast Asia

**DOI:** 10.3389/fvets.2024.1425394

**Published:** 2024-06-25

**Authors:** Chia-Hui Hsu, Chih-Hsuan Yang, Andres M. Perez

**Affiliations:** ^1^Center for Animal Health and Food Safety, College of Veterinary Medicine, University of Minnesota, Minneapolis, MN, United States; ^2^Department of Mechanical Engineering, Iowa State University, Ames, IA, United States

**Keywords:** African swine fever, Google trends, Vietnam, the Philippines, Thailand, public health, surveillance, epidemiology

## Abstract

African Swine Fever (ASF) is a reportable disease of swine that causes far-reaching losses to affected countries and regions. Early detection is critically important to contain and mitigate the impact of ASF outbreaks, for which timely available data is essential. This research examines the potential use of Google Trends data as an early indicator of ASF outbreaks in Southeast Asia, focusing on the three largest swine producing countries, namely, Vietnam, the Philippines, and Thailand. Cross-correlation and Kullback–Leibler (KL) divergence indicators were used to evaluate the association between Google search trends and the number of ASF outbreaks reported. Our analysis indicate strong and moderate correlations between Google search trends and number of ASF outbreaks reported in Vietnam and the Philippines, respectively. In contrast, Thailand, the country of this group in which outbreaks were reported last, exhibits the weakest correlation (KL = 2.64), highlighting variations in public awareness and disease dynamics. These findings suggest that Google search trends are valuable for early detection of ASF. As the disease becomes endemic, integrating trends with other epidemiological data may support the design and implementation of surveillance strategies for transboundary animal diseases in Southeast Asia.

## 1 Introduction

African Swine Fever (ASF), a highly contagious viral disease affecting pigs and wild boar, has had significant impacts on pig populations and the swine farming economy globally (1). Although the ASF virus does not infect humans, the disease poses a serious problem for food security worldwide. Following the introduction of ASF into China in August 2018, the disease spread through the Southeast Asia region and by December 2023, 19 countries in Asia had reported cases of ASF in either domestic pigs or wild boars (2). ASF disrupted the swine industry of South East Asia and the disease was first reported in the top three swine-producing countries in the region (Supplementary Table S1), Vietnam, the Philippines, and Thailand, in February 2019, July 2019, and January 2022, respectively.

A prerequisite for the assessment of the effectiveness of control measures for infectious diseases, such as ASF, is the availability of timely data on disease spread. However, those data are not always collected or reported in time due to a variety of factors, including resource limitations, data infrastructure, field conditions and reporting systems (3, 4). Alternatively, it has been proposed that the results of web searches may be used as a proxy for monitoring disease presence or spread (5, 6). For example, Google Trends has been effectively used to forecast seasonal influenza outbreaks caused by Influenza Virus A in the United States (7) and various other countries, demonstrating a strong correlation and leading to proposals for an Internet surveillance system (8). Despite encountering limitations and complexities (9), Google Trends remains a freely accessible and user-friendly interface for epidemiological research on infectious diseases. Use of results of web searches as a proxy for disease spread may have an impact and application on South East Asia, a region that by 2021, had a population of 589 million individuals, with approximately 440 million (~75%) of them being active internet users. Vietnam, the Philippines, and Thailand have experienced a substantial increase in their online user base and digital consumer population. This surge can be attributed to the ongoing improvement of internet accessibility and infrastructure in the area, contributing to the sustained growth of online engagement and activity.

In this study, we aimed to compare the results of Google Trends with data on ASF spread in the top three swine producing countries of South East Asia (Thailand, Vietnam, and the Philippines). Because ASF was introduced into the region as an emerging disease, the hypothesis here is that online search volume may have a relation with the number of outbreaks within a country or a specific region. During the initial introduction of the disease into a country we expected a corresponding increase in Google searches as people seek information about the situation. Considering that ASF impacts not only swine farmers but also significantly affects pork prices and the swine supply chain, we expect a concurrent rise in search activity during severe ASF outbreaks.

The results presented in this study will help to understand the history of ASF spread in the region. Results also provide evidence for the use of the methodology to the monitoring of disease spread, which may have broader applications for transboundary animal disease surveillance in the region and globally, contributing to the ultimate goal of mitigating the impact of emerging animal diseases worldwide.

## 2 Materials and methods

### 2.1 Outbreak data collection and processing

ASF outbreak data from the Philippines, Vietnam, and Thailand was obtained from the Food and Agriculture Organization’s EMPRES Global Animal Disease Information System (EMPRES-i).^1^ Specifically, a total of 52 weeks of outbreak data subsequent to the first reporting of the disease on each country were retrieved and extracted from the system.

### 2.2 Google trend data

For secondary data or predictor, Google Trends^2^ was sourced in this research. Google Trends distinguishes between search terms and search topics. Search terms are specific queries and their relative search volume within a given language, while search topics encompass a broader range of related terms irrespective of language. In our study, we adopted a “search topic selection” approach, integrating local languages to compare search patterns across different regions.

Google Trends offers insights into the volume of searches for particular keywords, providing an indication of the attention given to those topics in various countries. Concerning the African Swine Fever outbreak, keywords such as “African Swine Fever,” “African Swine Fever Virus,” and related terms were analyzed in local languages. Search topics were examined in both Vietnamese and English in Vietnam, in Thai and English in Thailand, and exclusively in English in the Philippines. Commonly used keywords revolved around “African Swine Fever” or “African Swine Fever Virus,” with some users employing suggested terms such as “Swine Flu” or “*dịch tả lợn châu phi*” (Vietnamese for African Swine Fever). This reflects the varied search behaviors within each country. Keywords exhibiting significant peaks in search volume were selected for further investigation, while those with inadequate data were disregarded.

The trend data collection timeframe was tailored according to the date of each country’s first officially confirmed case of ASF from EMPRES-i data. A period of 52 weeks before and after the initial detection was chosen to capture trends over time effectively. We analyzed up to three keywords, with the data normalized relative to the highest point of interest on the chart for the respective region and time frame. A score of 100 denotes peak popularity, while a score of 0 indicates insufficient data.

### 2.3 Statistical analysis

The ASF outbreak data and Google Trends data were aggregated on a weekly basis and juxtaposed for comparative analysis. To ensure synchronization of the timeline, date format was standardized according to the ISO week numbering system. Cross-correlation analysis was employed to measure the association between two signals or datasets—in this case, between Google Trends data and weekly African Swine Fever outbreak reports. This analysis helps to identify how the changes in one signal correspond to those in the other as they shift in time. In the context of our study, which focuses on the relationship between two time series (X and Y), it is posited that the Y series may be influenced by past time points of the X series. The sample cross-correlation function was utilized to identify lagged values of the X variable that could potentially predict changes in Y. Each lag represents a one-week interval in the results.

Kullback–Leibler (KL) divergence is a statistical measure for quantifying the difference between an arbitrary probability distribution and a reference probability distribution (10, 11). When interpreting the KL divergence value, a result of 0 indicates that the two distributions in question are identical. Conversely, an increase in divergence signifies that the arbitrary probability distribution deviates more from the reference distribution. Therefore, smaller KL divergence values are desired when the goal is to approximate the reference probability distribution closely with a predictive distribution.

The statistical analyses were conducted using R version 4.2.2. The similarity and possible time shifts between datasets were assessed with the ccf() function, which performs cross-correlation analysis. The KL divergence was calculated using the scikit-learn library, and the data was visualized by Python’s Matplotlib.

## 3 Results

### 3.1 ASF outbreak and Google trend correlation in Southeast Asia

**Figure 1:**
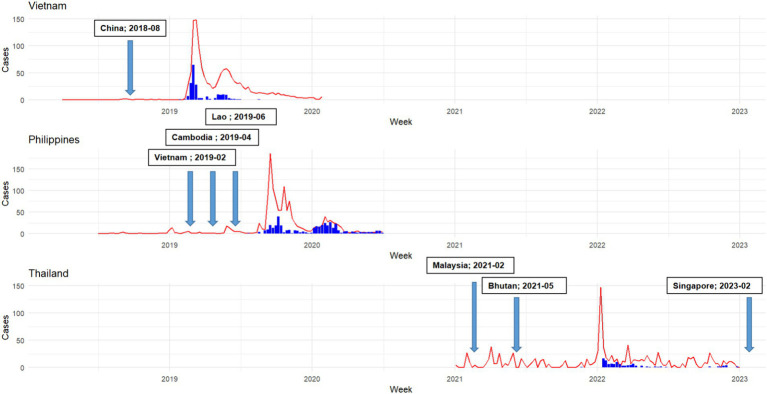
African Swine Fever (ASF) outbreaks (shown as blue bars) and Google Trends search volume for selected ASF-related keywords (represented by red lines) from 2018 to 2023 in Vietnam, Philippines, and Thailand. The arrows indicate when neighboring countries first reported ASF to the World Organization for Animal Health (WOAH). In the case of Vietnam and the Philippines, the timing of peaks in Google search volume (red line) aligns with peaks in ASF outbreak cases (blue bars), suggesting a synchrony between online search activity and the occurrence of ASF outbreaks in the field.

In both Vietnam and the Philippines, a significant alignment was found between the epidemic curve and Google trends results (Figure 1). In Vietnam, the ASF outbreak began in Week 5 of 2019 (February) and peaked in Week 10. A secondary peak occurred during Weeks 19 and 20 (May 2019). In the Philippines, the first ASF outbreak was recorded in Week 29 (July 2019), reaching its highest point in Week 40 (October 2019). However, in the descriptive analysis for Thailand, before the initial detection of ASF, the Google trend curve showed numerous smaller peaks. This pattern contrasts with the trends observed in Vietnam and the Philippines.

### 3.2 Cross-correlation and KL divergence results in Southeast Asia

**Figure 2:**
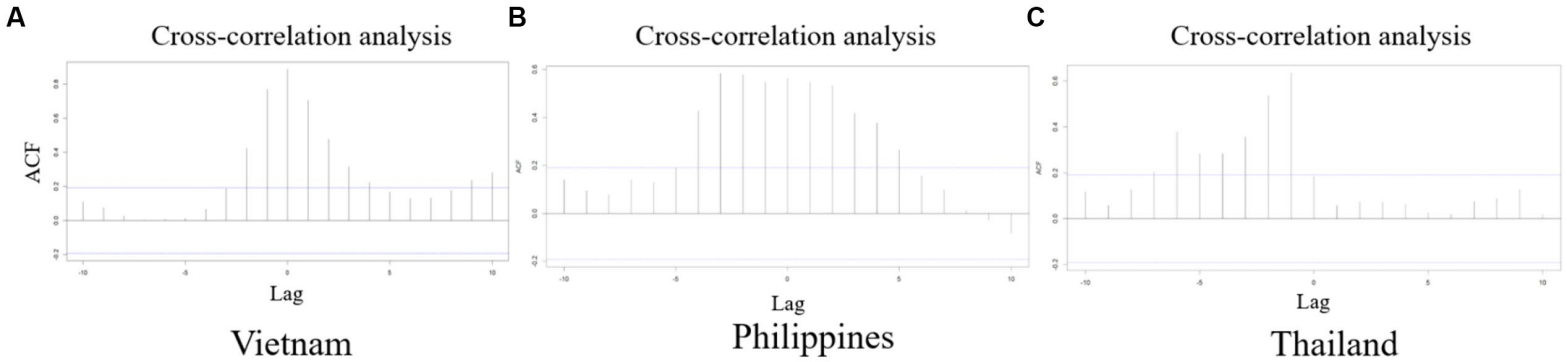
This figure presents the cross-correlation analysis between Google Trends search data and disease outbreaks. In the cross-correlation plot for Vietnam **(A)**, a significant correlation is indicated by peaks that exceed the blue dashed threshold line. A lag of 0 suggests a simultaneous peak in search data and ASF outbreaks, indicating a meaningful positive correlation. Additional peaks at lag −2 and lag 2 also indicate significant correlations. In the Philippines **(B)**, significant correlations are identified at lag −4 and lag 5, with the most prominent peak at lag −3. These peaks exceed the blue dashed threshold line, indicating a strong connection between Google Trends data and ASF outbreak timing. For Thailand **(C)**, no significant correlation is found at lag 0. However, there is notable correlation between lag −1 and lag −6, with the strongest peak at lag −1, suggesting a possible time shift between search trends and disease outbreaks.

**Figure 3:**
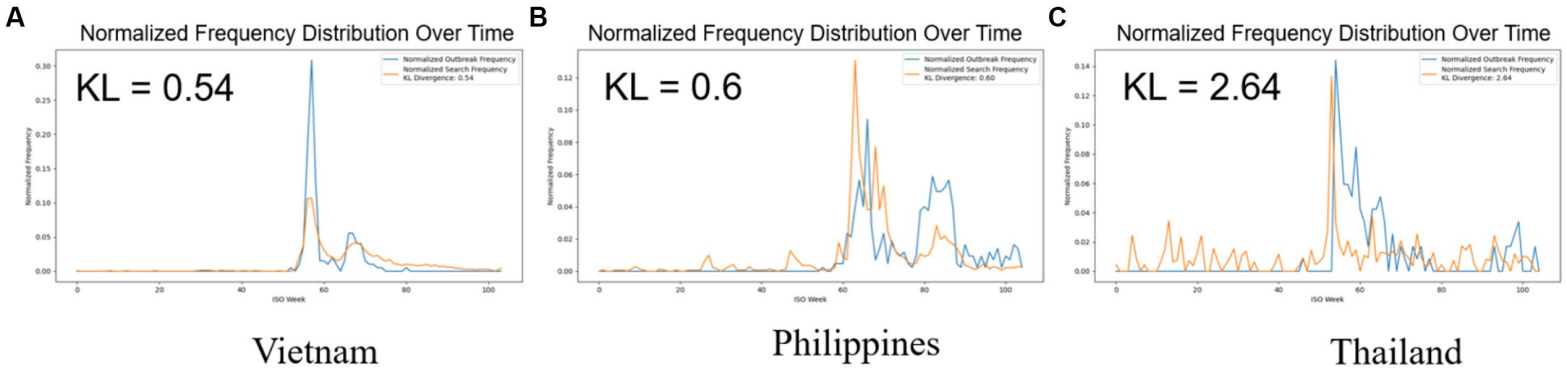
This figure uses KL Divergence to compare normalized ASF outbreak and Google search frequencies across three Southeast Asian countries. The blue lines represent ASF outbreaks, while the orange lines represent Google search frequencies. It displays the KL divergence values of the normalized search frequency with the reference to the normalized search frequency, with respective. The KL divergence quantifies the discrepancy between the two distributions, where a lower value indicates higher similarity. Results are as follows: **(A)** Vietnam demonstrates the closest alignment (KL divergence: 0.54), **(B)** Philippines shows moderate congruence (KL divergence: 0.6), and **(C)** Thailand exhibits the largest divergence (2.64), indicating less correlation.

The cross-correlation analysis (Figure 2) provided insights into the time-lagged relationships between Google Trends search data and ASF outbreak in Southeast Asia. It highlighted the correlation peaks for different lags, suggesting patterns of synchronicity or shifts between search behavior and disease outbreaks. KL Divergence analysis (Figure 3) offered a quantifiable measure of the congruence between these two datasets.

Vietnam demonstrated the strongest correspondence between Google search trends and ASF outbreaks (KL = 0.54), indicating a close alignment between search behavior and outbreak patterns. The Philippines showed a similar pattern with a notable correlation (KL = 0.6). In contrast, Thailand exhibited the least correlation (KL = 2.64), suggesting its search behavior is less aligned with outbreak occurrences. These results reveal differences in search behavior and ASF outbreak trends across Southeast Asia.

## 4 Discussion

This research is the first to connect ASF outbreaks with Google Trends data, demonstrating that this tool is accessible and reproducible for analysis across various regions and languages in Southeast Asia. The results of the cross-correlation and quantifiable KL divergence analyses suggest that Google Trends can offer meaningful insights into patterns of public concern related to ASF.

Our analysis reveals varying levels of performance disparities among Vietnam, the Philippines, and Thailand. Vietnam demonstrates the most robust performance, with a lag of 0 and the lowest KL divergence, indicating a high degree of synchronization between the two datasets. Vietnam experienced the first ASF outbreak in Southeast Asia in February 2019, and as a new transboundary animal disease, public awareness of ASF was initially limited. This lack of awareness may have played a role in establishing a direct or indirect correlation between the severity of the outbreak and Google search volume. A similar pattern emerged in the Philippines, which also showed a low KL divergence value, with the ASF outbreak beginning in July 2019, about six months after the initial outbreak in Vietnam. This parallel timing suggests a comparable trend in public awareness and Google search activity. However, as the ASF outbreak rapidly spread to Thailand and other Southeast Asian countries, entering its third year in the region, the dynamics shifted. Regional familiarity with the disease likely escalated, leading to a nuanced understanding of its awareness. This evolving awareness may elucidate the presence of peak noise in Google Trend results depicted in Figure 2 for Thailand, with an interval of 1 to 6 weeks difference.

ASF outbreaks in Vietnam and the Philippines were believed to have been mainly linked to the importation of pork products or tourists carrying items containing the genotype II ASF virus (12, 13). However, the transmission route in Thailand may have been different, likely related to owners importing pigs as companion animals. These imported pigs were later diagnosed with ASFV (14). On December 2021, the pig owner sent the carcass of one of the pigs to Kasetsart University for investigation into the sudden death. Subsequently, in January 2022, Thai authorities officially confirmed the presence of ASFV in the country, prompting the initiation of active and passive surveillance measures comprehensively (14). Currently, the spreading investigation from companion pigs to domestic farms and the transmission mechanism during the early stages of the ASF epidemic in Thailand remain unclear. There is also suspicion that the virus may have emerged earlier than indicated by official reports from Thailand authorities.

Our analysis of Thailand revealed a notable trend in Google searches: multiple search surges occurred before the official confirmation of the ASF outbreak in January 2022, indicating a potentially earlier occurrence than reported. While this observation alone may not be decisive, it raises questions about the timing of the first outbreak. Further evidence from Taiwanese airport surveillance showed that PCR testing detected ASFV in pork products from Nakhon Pathom province, Thailand, as early as September 30, 2021 (15). This molecular evidence strongly implied the possible infiltration of ASFV into Thailand’s pork supply chain before official confirmation through local swine farm sampling. Integration of Google Trends methodology could potentially offer valuable insights into improving early detection efforts.

Using Google Trends to monitor disease outbreaks has its benefits and limitations. It can be a useful tool for gauging public concern in real-time during the early stages of an outbreak. Our analysis indicates that cross-correlation with Google search data works best when a transboundary animal disease is new to a specific region, providing an effective predictive or indicative index. However, as a disease like ASF becomes endemic, the effectiveness of this approach may decrease, as the heightened public interest wanes over time. This underscores the need for flexible approaches when using search data for epidemiological surveillance and suggests that other strategies should be used alongside Google Trends to sustain relevance throughout the different phases of disease outbreak.

Several limitations of using Google Trends for disease outbreak analysis need to be considered. Although our methodology aims to reduce translation issues, direct translations can lead to a loss of subtlety. User search habits and behavior can vary, affecting search volume, and external factors such as news events, government policies, or marketing campaigns can influence public search patterns. Disparities in internet access and digital literacy can also introduce biases, potentially amplifying noise in urban areas and diminishing the signal in rural regions. Given these limitations, a surge in search activity might alert epidemiologists, but the data must be contextualized with other epidemiological information for a comprehensive understanding of ASF dynamics.

Lastly, our research is inherently biased by focusing on Google Search as the primary search engine in Southeast Asia. Exploring other search engines or locally popular social media platforms could offer additional insights. Studies have shown that platforms like Twitter can offer better precision in tracking diseases like the influenza virus through mathematical models in Greece (16). Therefore, exploring alternative competitive platforms or locally commonly used social media could enhance the effectiveness of disease outbreak analysis for future research directions.

In conclusion, our research demonstrates that comparing Google Trends data with official ASF outbreak data can reveal patterns of public interest that correspond to ASF outbreaks. This methodology, especially in a new region with emerging transboundary diseases, showed a significant correlation, suggesting that Google Trends could potentially serve as an early indicator for ASF outbreaks. However, to gain a comprehensive understanding of ASF dynamics, Google Trends data should be used in conjunction with other epidemiological information and interpreted with caution. This study highlights the value of flexible monitoring approaches and the need for further research into factors affecting public awareness and ASF outbreaks.

## Data availability statement

The original contributions presented in the study are included in the article/Supplementary material, further inquiries can be directed to the corresponding author.

## Author contributions

C-HH: Conceptualization, Data curation, Formal analysis, Investigation, Methodology, Project administration, Software, Visualization, Writing – original draft, Writing – review & editing. C-HY: Investigation, Methodology, Software, Visualization, Writing – review & editing. AP: Conceptualization, Funding acquisition, Methodology, Resources, Supervision, Writing – review & editing.

## Footnotes

^1^https://empres-i.apps.fao.org/

^2^https://trends.google.com/trends/
