# Supplementary material for: Google trends as an early indicator of African swine fever outbreaks in Southeast Asia
Source: Front Vet Sci. 2024 Jun 25;11:1425394. doi: 10.3389/fvets.2024.1425394 (PMC11231385; doi:10.3389/fvets.2024.1425394)
Supplement: Supplementary file 1 [file Table_1.DOCX]

**Supplementary A**

**ASF outbreak Southeast Asian countries from 2018-2023 (In chronological order):**

| No. | Country | First ASF date | Swine population^1^ | Annotation | Human population^2^ (M)  [(Y2020)](https://www.statista.com/statistics/796222/total-population-of-the-asean-countries/#:~:text=In%202021%2C%20the%20total%20population,Singapore%2C%20Thailand%2C%20and%20Vietnam.) | [Percentages of individuals using the internet](https://data.worldbank.org/?locations=TH-MY-ID-SG-PH-VN) | Online population estimate (M) |
| --- | --- | --- | --- | --- | --- | --- | --- |
| 1 | Vietnam | 19 February 2019 | 22,027,858 | Official figure | 97.58 | 79% | 77.09 |
| 2 | Cambodia | April 2019 | 2,108,052 | Estimated value | 15.68 | 60%* | 9.41 |
| 3 | Laos | 20 June 2019 | 4,298,000 | Official figure | 7.32 | 62%* | 4.54 |
| 4 | The Philippines | July 2019 | 12,795,721 | Official figure | 108.77 | 53% | 57.65 |
| 5 | Myanmar | August 2019 | 6,778,694 | Official figure | 53.2 | 44%* | 23.41 |
| 6 | Malaysia | February 2021 | 1,869,772 | Official figure | 32.45 | 97% | 31.48 |
| 7 | Thailand | January 2022 | 7,727,959 | Imputed value | 69.8 | 88% | 61.42 |
| 8 | Singapore | February 2023 | 0 | no domestic pig production | 5.69 | 96% | 5.46 |
| ^1^Source from FAOSTAT  ^2^Source from Statista. Total population of the Association of Southeast Asian Nations (ASEAN) countries from 2018 to 2028  *Source from the World Bank, Year of 2021 | | | | | | | |

**Supplementary B**

**Kullback-Leibler (KL) divergence equation:**

**
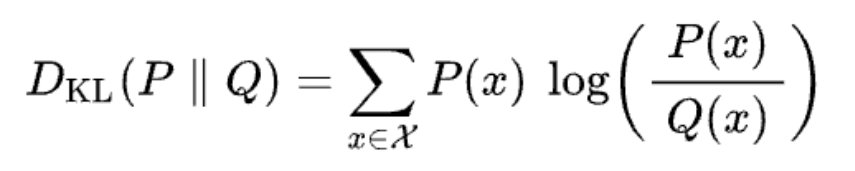
**

where 𝑝(𝑥) and 𝑞(𝑥) represent the frequencies per week.

- 𝑥: the x-th week
- 𝑝(𝑥): the frequency of outbreaks in the x-th week divided by the total number of outbreaks
- 𝑞(𝑥): the frequency of searches in the x-th week divided by the total number of searches

**KL divergence Steps**

To compute the Kullback-Leibler (KL) divergence between two distributions *p* and *q*, where *p* represents the outbreak frequency per week and *q* represents the search frequency per week, the process involves several steps.

1. Define the **kl_divergence** function, which takes *p* and *q* as inputs.
2. Normalize the distributions so that the elements of *p* sum to 1 and the elements of *q* sum to 1. For each element in *p*, if the element is non-zero, compute *p*[*i*]×log(*p*[*i*]/*q*[*i*]); if the element in p is zero, contribute 0 to the sum to handle the 0log(0/*q*) case.
3. Sum all the computed values to get the final KL divergence using ∑*p*[*i*]log(*p*[*i*]/*q*[*i*]).
4. Return the computed KL divergence value.

**Python Code:**

# p(x) is outbreak; q(x) is search

import pandas as pd

import numpy as np

import matplotlib.pyplot as plt

from scipy.stats import entropy

def plot_normalized_time_series(df, name, save_path):

"""

Plots normalized time series data for 'Search' and 'Outbreak' columns in a DataFrame,

where each column's values are scaled to sum up to one, computes the KL divergence,

using 'Outbreak' as the ground truth.

Parameters:

- df: pandas DataFrame with a datetime index.

- name: A string to name the output plot file.

- save_path: Path to save the output plot.

"""

column1 = 'Search'

column2 = 'Outbreak'

# Normalize the columns

df_normalized = df[[column1, column2]].apply(lambda x: x / x.sum(), axis=0)

# Compute KL divergence with Outbreak as ground truth

kl_div = entropy(df_normalized[column2] + 1e-10, df_normalized[column1] + 1e-10)

plt.figure(figsize=(12, 6))

plt.plot(df_normalized.index, df_normalized[column2], label=f'Normalized {column2} Frequency')

plt.plot(df_normalized.index, df_normalized[column1], label=f'Normalized {column1} Frequency \nKL Divergence: {kl_div:.2f}')

plt.xlabel('ISO Week')

plt.ylabel('Normalized Frequency')

plt.title(f'Normalized Frequency Distribution Over Time for {name}')

plt.legend()

plt.savefig(f'{save_path}{name}_normalized_time_series.png')

plt.close()

# Define the path to the file

file_path = '/Users/tcpba/2024Spring/Jesper_research/raw_data.xlsx'

save_path = '/Users/tcpba/2024Spring/Jesper_research/results/'

# Reading all sheets at once

xlsx = pd.read_excel(file_path, sheet_name=None, engine='openpyxl')

# xlsx is a dictionary. To access each DataFrame, use the sheet name as the key.

df_T = xlsx['Thailand'] # Replace 'Sheet1' with the actual name of your first sheet.

df_P = xlsx['Philippines']

df_V = xlsx['Vietnam'] # Adjust the names as necessary.

# Display the first few rows of the DataFrame to verify it's loaded correctly

for country in ['Thailand','Philippines','Vietnam']:

print('country',country )

df = xlsx[country]

plot_normalized_time_series(df,name = country,save_path = save_path)

**Cross-correlation function**

The ccf() function in R computes the cross-correlation function between two time series, 𝑝 (𝑥) and 𝑞(𝑥), which represent the outbreak and search time series, respectively. The cross-correlation function measures the similarity between the two time series as a function of the lag 𝑘.


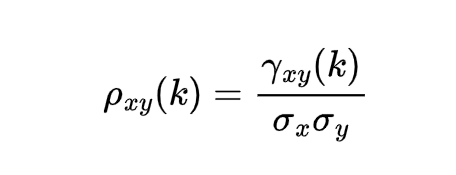


where:

- ρ_xy_(k) is the cross-correlation at lag k.
- γ_xy_​(k) is the cross-covariance at lag k.
- σ_x_ ​is the standard deviation of x.
- σ_y_ is the standard deviation of y.
